# The evolution of FLASH radiotherapy: a bibliometric analysis

**DOI:** 10.3389/fonc.2025.1580848

**Published:** 2025-05-15

**Authors:** Shuokai Jia, Weige Wei, Yiwen He, Haichuan Yan, Shiru Zou, Yanmei Hao, Qing Xiao, Guangjun Li

**Affiliations:** ^1^ Department of Radiation Oncology, Cancer Center, West China Hospital, Sichuan University, Chengdu, Sichuan, China; ^2^ Department of Radiotherapy Physics & Technology, West China Hospital, Sichuan University, Chengdu, Sichuan, China

**Keywords:** FLASH radiotherapy (FLASH-RT), ultra-high dose rate (UHDR), biological mechanisms, device, preclinical and clinical trials, bibliometric analysis

## Abstract

**Introduction:**

FLASH radiotherapy (FLASH-RT) represents a groundbreaking technique, characterized by its ultra-high dose rate and its remarkable ability to spare normal tissues from damage. Numerous studies on FLASH-RT have been conducted worldwide. However, to date, no comprehensive bibliometric analysis has been performed in this field. This study aims to provide an overview of the advancements in FLASH-RT and identify potential future research directions through bibliometric analysis.

**Method and materials:**

The research team performed a literature search in the Web of Science Core Collection (WOSCC), covering the period from 1967 to 2024, and identified 461 publications relevant to the field of interest. Visualization tools, including VOSviewer, CiteSpace, and Bibliometrix, were employed to analyze countries, institutions, authors, journals, references, and keywords, thereby uncovering research frontiers and hotspots within the field.

**Results:**

In recent years, a considerable number of publications on FLASH-RT have emerged. The United States has the highest number of publications (n=208). The institution with the highest publication count is “Lausanne University Hospital” (n=39). The author with the most citations is “Vozenin, M” (n=31), while the author with the most co-citations is “Montay-Gruel, P” (n=812). Medical Physics is the journal with the highest number of both publications and co-citations, whereas Radiotherapy and Oncology has the highest number of citations. The paper titled “Ultrahigh dose-rate FLASH irradiation increases the differential response between normal and tumor tissue in mice” has the highest number of both citations and co-citations. The most frequently co-occurring keywords is “FLASH radiotherapy” (n=379).

**Conclusions:**

Our bibliometric analysis of FLASH-RT explores key dimensions of the field, including publication trends, international collaborations, influential journals and authors, and keyword evolution. It assesses FLASH-RT’s historical development, current global status, and recent progress in biological mechanisms, equipment, and clinical translation, aiming to offer researchers a comprehensive overview.

## 1 Introduction

In recent years, cancer has become a major global health challenge, with over half of all patients requiring radiation therapy during treatment (1, 2). This approach utilizes high-energy radiation, including photons, electrons, protons and heavy ions, to damage the DNA of cancer cells, thereby inhibiting their growth and survival (3, 4). However, radiation may also affect surrounding healthy tissues, potentially causing toxicity and long-term complications, highlighting the need for careful management to minimize these risks (5).

For decades, the primary goal of radiotherapy has been to maximize tumor control while minimizing damage to normal tissues, thereby reducing radiation-induced complications. High-energy radiation at conventional dose rates causes damage to tumors while also causing injury to normal tissues. In recent years, advancements in technologies such as Intensity-Modulated Radiation Therapy, Image-Guided Radiation Therapy, particle therapy, and Stereotactic Body Radiation Therapy (SBRT) have considerably improved the therapeutic outcomes (6, 7). Current research is increasingly focused on the development of innovative treatment modalities, with FLASH radiotherapy (FLASH-RT) emerging as one of the most promising techniques (8).

FLASH-RT, characterized by its ultra-high dose rate (UHDR) delivery within an extremely short duration, is believed to induce the “FLASH effect”, which enhances normal tissue tolerance while maintaining high therapeutic doses to tumors (9, 10). Numerous studies have demonstrated that FLASH-RT can mitigate toxicity to normal tissues while achieving therapeutic outcomes comparable to conventional radiotherapy (11–14).

The advantages of FLASH-RT have drawn significant scientific interest, sparking global discussions and expanding research efforts. These studies highlight current trends, key characteristics, and the challenges to its sustainable development. Identifying key studies that guide FLASH-RT, support its clinical implementation, and predict future research trends is crucial for advancing this promising modality.

Previous retrospective studies have summarized FLASH-RT development, often focusing on specific aspects like radiobiological mechanisms, beam type development, or historical milestones (15–17). Bibliometric analysis, using quantitative methods to identify patterns in knowledge dissemination, offers a scientific approach to exploring trends and future directions within a field. This systematic research approach has been widely applied in radiotherapy. Currently, researchers have conducted bibliometric studies on several radiotherapy techniques, such as proton therapy (18) and SBRT (19). Additionally, bibliometric analyses related to radiotherapy focusing on specific cancer sites, including nasopharyngeal carcinoma (20) and rectal cancer (21). However, to the best of our knowledge, no dedicated bibliometric analysis focusing specifically on FLASH-RT has been reported to date. As the first comprehensive bibliometric study on FLASH-RT, this research aims to map its mechanisms, technological advancements, and clinical translation, providing valuable insights to guide its development and future investigations.

## 2 Materials and methods

### 2.1 Data source search strategy

The Web of Science Core Collection (WOSCC) serves as a crucial database for accessing a comprehensive range of global academic information. Highly regarded within the academic community, this database acts as a wealth of academic resources for researchers. For this analysis, the Science Citation Index Expanded (SCI-E) database was chosen as the main data source. On September 23, 2024, a comprehensive search and update of references was performed within the SCI-E, which is particularly well-suited for bibliometric analysis (22). Literature retrieval was completed within one day to avoid citation fluctuations caused by the rapid update of publications.

**Figure 1:**
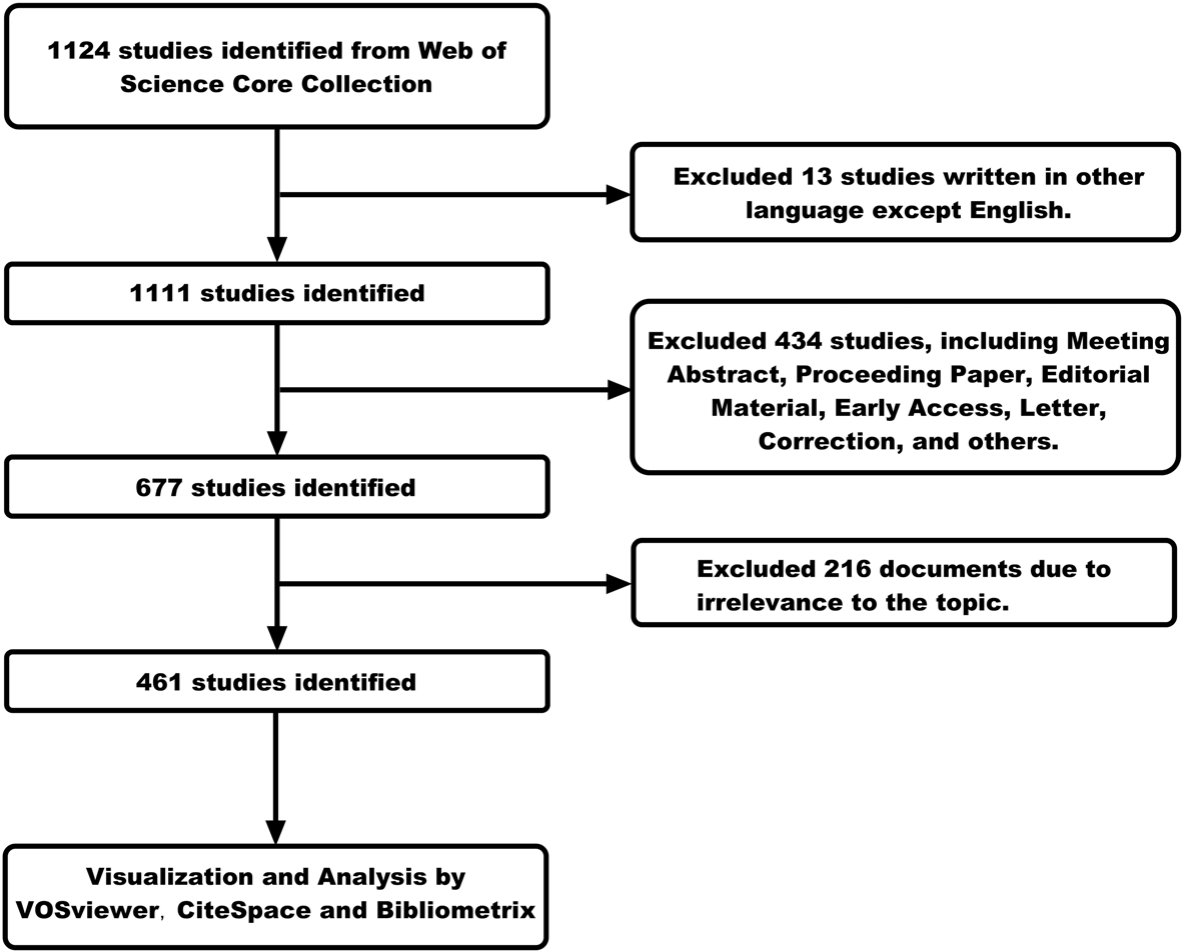
Publications screening flowchart.

The key search terms included “FLASH”, “ultra-high dose rate”, “radiation therapy”, “radiotherapy”, “clinical trials”, “clinical translation”, “LINAC”, “dose delivery”, “proton therapy”, and “particle therapy”. We have arranged and combined these search terms to develop a rigorous search strategy. We searched and retrieved data on FLASH-RT from the past few decades (from 1967 to 2024), obtaining 1,124 publications. Only publications in the English language were included in this study (n=1111). Our study included only “articles” and “review” (n=677). “Meeting Abstract”, “Proceeding Paper”, “Editorial Material”, “Early Access”, “Letter”, “Correction”, and other types of publications were not considered. Then, we read the titles, abstracts, and even full texts of these publications to screen out papers that were closely related to our research topic, ensuring the purity of the data. Ultimately, only 461 publications were included in the bibliometric analysis. A detailed description of the search strategy can be found in the **Supplementary Material**, while the screening methodology is outlined in **Figure 1**.

### 2.2 Data analysis and visualization

In this research, we utilized VOSviewer (1.6.20), CiteSpace (5.7.R2), and Bibliometrix (4.4.1) to analyze and visualize 461 academic articles. Data on authors, nationalities, affiliations, article titles, journals, citation counts, and keywords were collected for comprehensive analysis.

VOSviewer, a free and open-source software, is highly effective in visualizing bibliometric networks, including relationships between keywords, authors, and publications (23). The counting method adopts “Full counting”. The normalization method uses the option “Association strength”. The visualization layout employs the “Force Atlas 2 algorithm” to achieve optimal cluster formation. In the results of visual analysis: the color of nodes represents different clusters, the size of nodes indicates the weight of the node, and the thickness of lines represents the strength of the relationship.

CiteSpace identifies research hotspots, emerging trends, and knowledge structures through co-citation and citation burst analyses. We employed the g-index as the literature selection criterion. The network scale was controlled by setting the parameter k=25 in the selection criteria. For burst detection analysis, we adjusted the “Minimum Duration” to 1 to capture emerging trends while maintaining sensitivity to short-term scholarly attention. We adjust the γ parameter (gamma value) to keep the number of “Burst Items” within a reasonable range. For the visualization analysis of CiteSpace, the red bar graph represents the frequent occurrence of keywords, while the blue bar graph indicates infrequent occurrence of keywords. The greater the intensity of the bar graph, the higher the frequency of occurrence.

Bibliometrix, an R-based software, offers extensive bibliometric analysis capabilities, including performance metrics and science mapping across various databases (24). The retrieved 461 articles were exported in BibTeX format to R environment. The exported files contained citation metadata including authors, titles, journals, institutions, etc. Bibliometric diagrams were generated using the bibliometrix R package. The “summary()” function was employed to analyze the most relevant source and country scientific production. The visualization analysis results presented by Bibliometrix indicate that countries marked in blue represent those with national scientific output. The depth of the blue color is proportional to the national scientific output, with darker blue signifying higher national scientific output.

## 3 Results

### 3.1 Publication outputs and time trend

**Figure 2:**
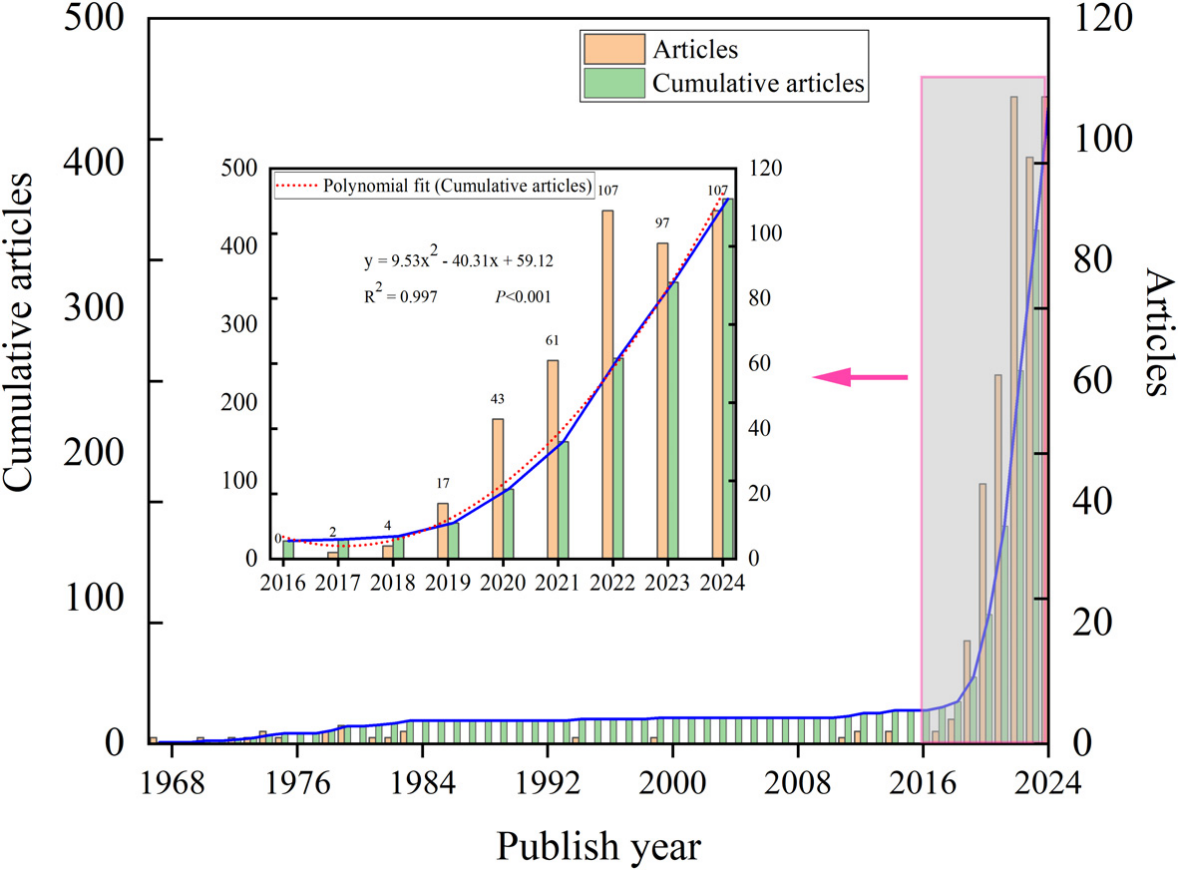
Global publication trends on FLASH-RT.

The publication trend of FLASH-RT from 1967 to 2024 can be divided into two distinct phases. Initial reports on FLASH date back to 1967. Between 1967 and 2017, publications were sporadic, with a fragmented distribution and several years without relevant articles. In contrast, a marked increase in annual publications has been observed from 2017 to 2024 (**Figure 2**). To analyze this recent surge, a mathematical model was applied to fit the publication trend. The mathematical model employed in our analysis is y = 9.5292x²- 40.309x + 59.119 (R²= 0.9973, P<0.001) (**Figure 2**). Data collection was completed on September 23, 2024, and it is anticipated that the actual number of publications for 2024 will surpass the number reported in the literature.

### 3.2 Distribution of country and institution

**Figure 3:**
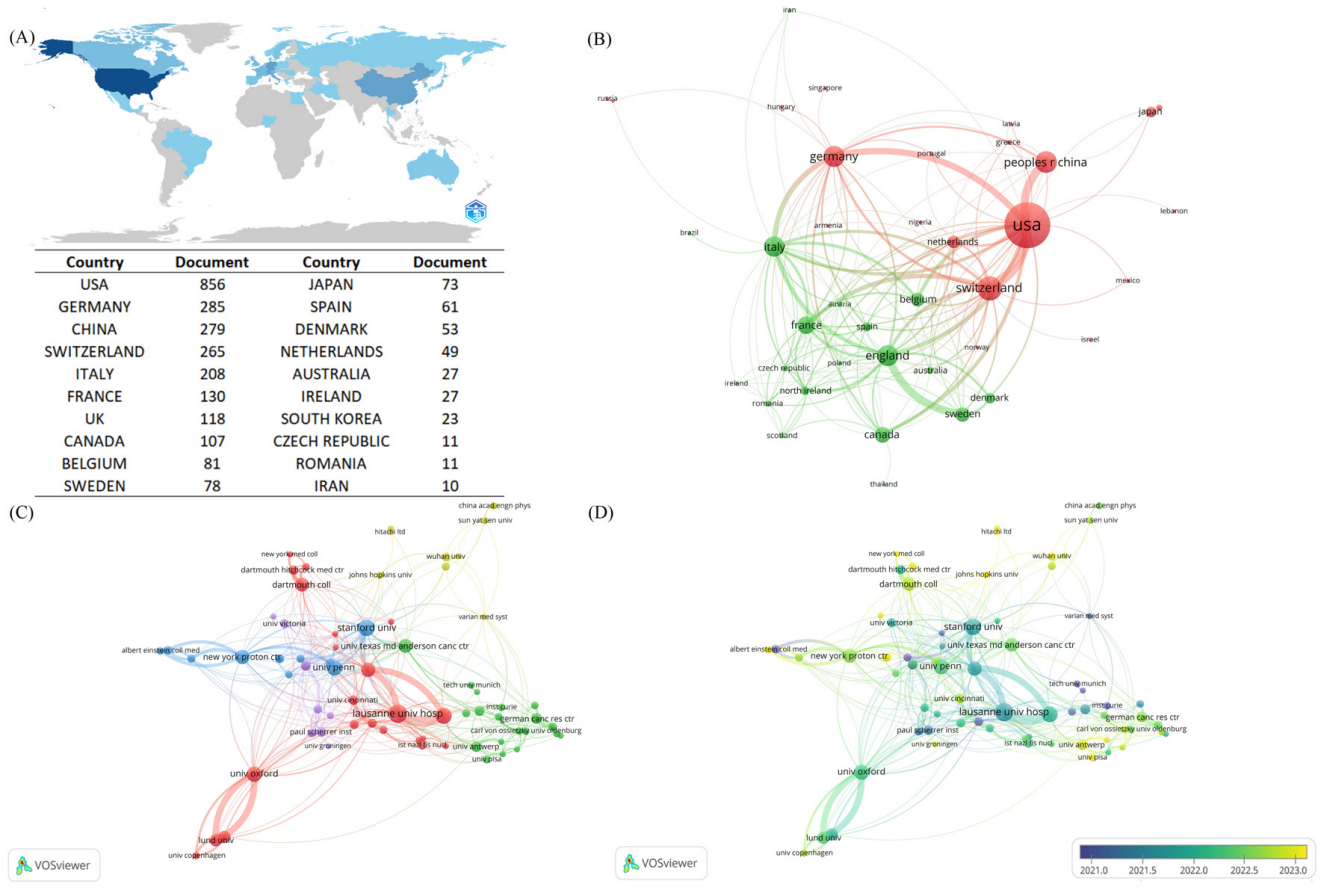
Contributions and co-operative relations between countries and institution. **(A)** Countries marked in blue in the figure represent countries with national scientific production in the FLASH-RT neighborhood, and the degree of blue is proportional to national scientific production, with darker blue representing higher national scientific production. Specific national science production values are also presented in this figure. **(B)** Map of country co-operation. Different colors represent different clusters. A node represents a country, the larger the node the greater the number of publications for that country. The lines between countries represent the co-operation between countries, the thicker the line the stronger the co-operation between countries. **(C)** Institutional co-authorship relationship map. Different colors represent different clusters. The size of the nodes is proportional to the number of publications of the institutions. The thicker the connecting line between the nodes indicates the closer the co-authorship between the two institutions. **(D)** Chart of average year of institutional issuance. Yellower colors indicate a later average year of institutional issuance.

A total of 40 countries have contributed to FLASH-RT from 1967 to 2024. Global scientific productivity was analyzed with Bibliometrix, revealing that the United States exhibits the highest level of scientific output in this area (**Figure 3A**). An analysis of international collaborations, conducted through VOSviewer, identified two distinct clusters of countries. One cluster, centered around the United States, includes close collaborations with China, Germany, and Switzerland. The other cluster, led by the United Kingdom, is closely associated with France, Italy, and Sweden (**Figure 3B**). The top five countries by publication volume are the United States (n=208), Switzerland (n=69), China (n=56), Germany (n=53), and the United Kingdom (n=52).

We also mapped the network of collaborations among institutions involved in FLASH-RT research. A total of 700 institutions worldwide contribute to this field, with 74 organizations publishing five or more articles. After excluding two isolated nodes, the remaining 72 institutions were grouped into five clusters (**Figure 3C**). “Lausanne Univ Hosp” has the highest number of publications (n=39) and exhibits the strongest co-authorship relationships with other institutions. Additionally, we plotted the average age of these institutions based on their years of publication activity, revealing a trend towards increasing institutional engagement in this research area over time (**Figure 3D**).

### 3.3 Co-authorship and co-citation between authors

**Figure 4:**
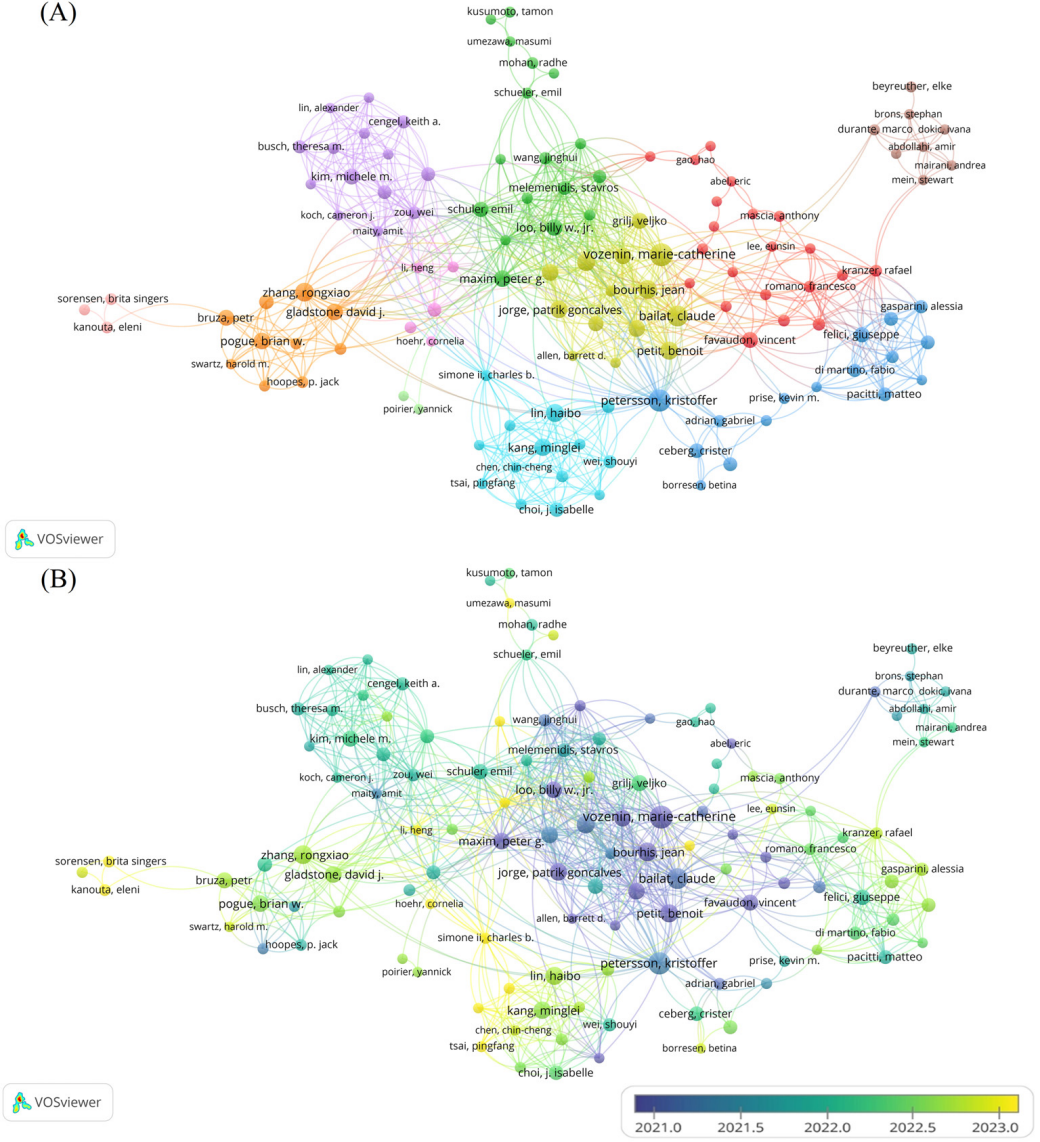
Analysis of the characteristics of the author’s posting. **(A)** Co-authorship between authors. Different colors represent different clusters. Each node represents an author, the larger the node, the more publications the author has. The line between authors represents the co-authorship relationship between authors, the thicker the line, the stronger the co-authorship relationship between authors. **(B)** Graph of the average number of years authors have been posting. Different colors represent different years of posting.

A total of 2,322 authors contributed to the field during the study period. We visualized the co-authorship relationships among authors with more than five publications (**Figures 4A, B**). After excluding a few isolated nodes, the co-authorship network of 124 authors was revealed, grouped into 11 clusters. The top five authors with the highest publication counts are Vozenin, M (n=31), Petersson, K (n=28), Bailat, C (n=24), Bourhis, J (n=20), and Zhang, R (n=19), all of whom have made significant contributions and exert considerable influence in the field. The co-authorship relationships within each cluster are strong, and there are also significant collaborative links between authors across different clusters.

**Figure 5:**
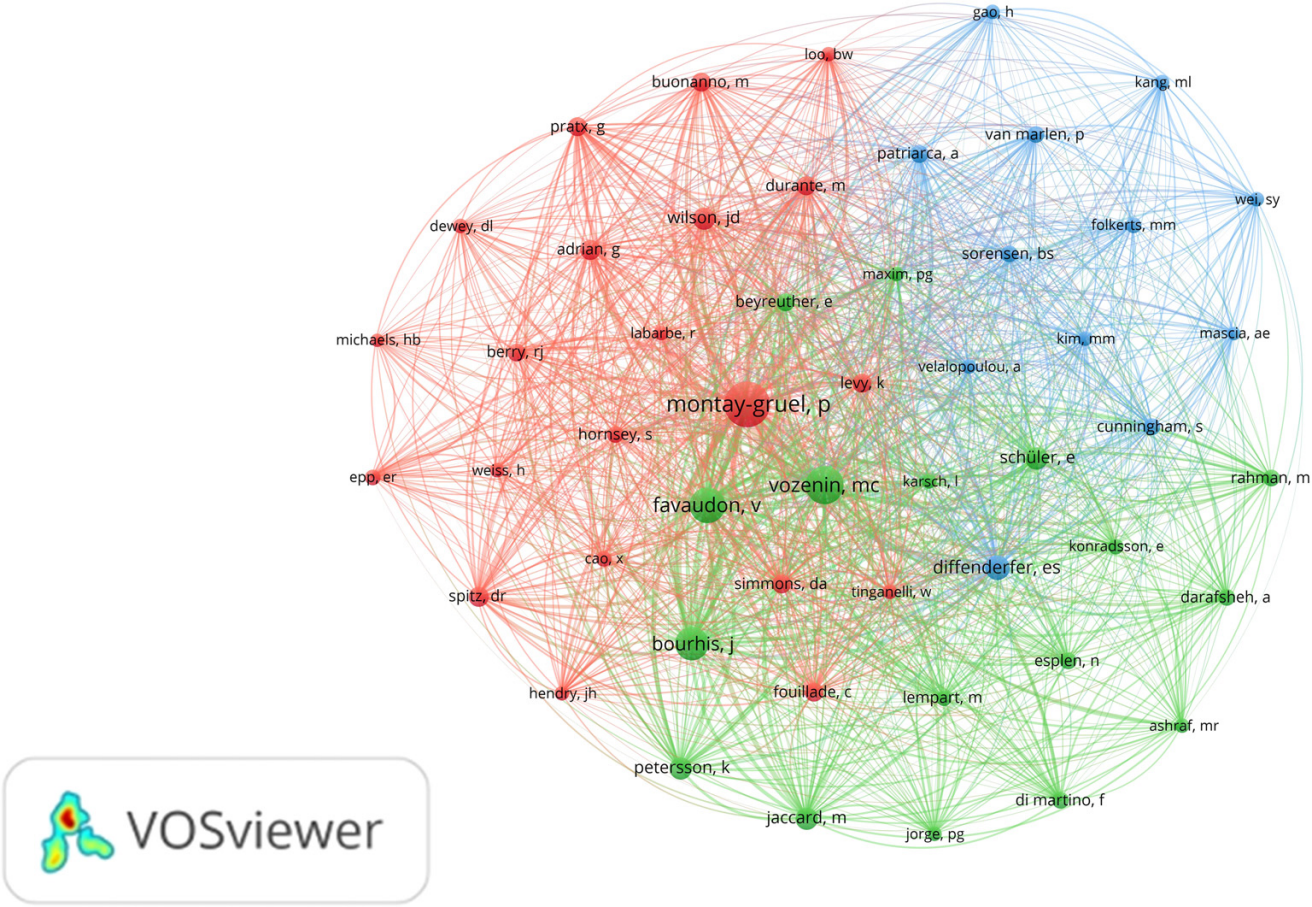
Co-citation analysis between authors. Different colors represent different clusters. Each node represents an author, and the larger the node, the more co-citations the author has. A line between different authors represents a co-citation relationship between authors, the thicker the line, the more co-citations between two authors.

The analysis of co-citations among authors is equally important. Among the 5,785 authors with co-citation relationships, 50 authors had a significantly higher co-citation count, each exceeding 60. A visual analysis of the co-citation relationships among these authors was conducted (**Figure 5**). The five authors with over 200 co-citations are Montay-Gruel, P (n=812), Vozenin, M (n=532), Favaudon, V (n=464), Bourhis, J (n=412), and Diffenderfer, E (n=217). These authors play a central role in the field, as evidenced by their high co-citation frequencies.

### 3.4 Visualization and analysis of journals

**Figure 6:**
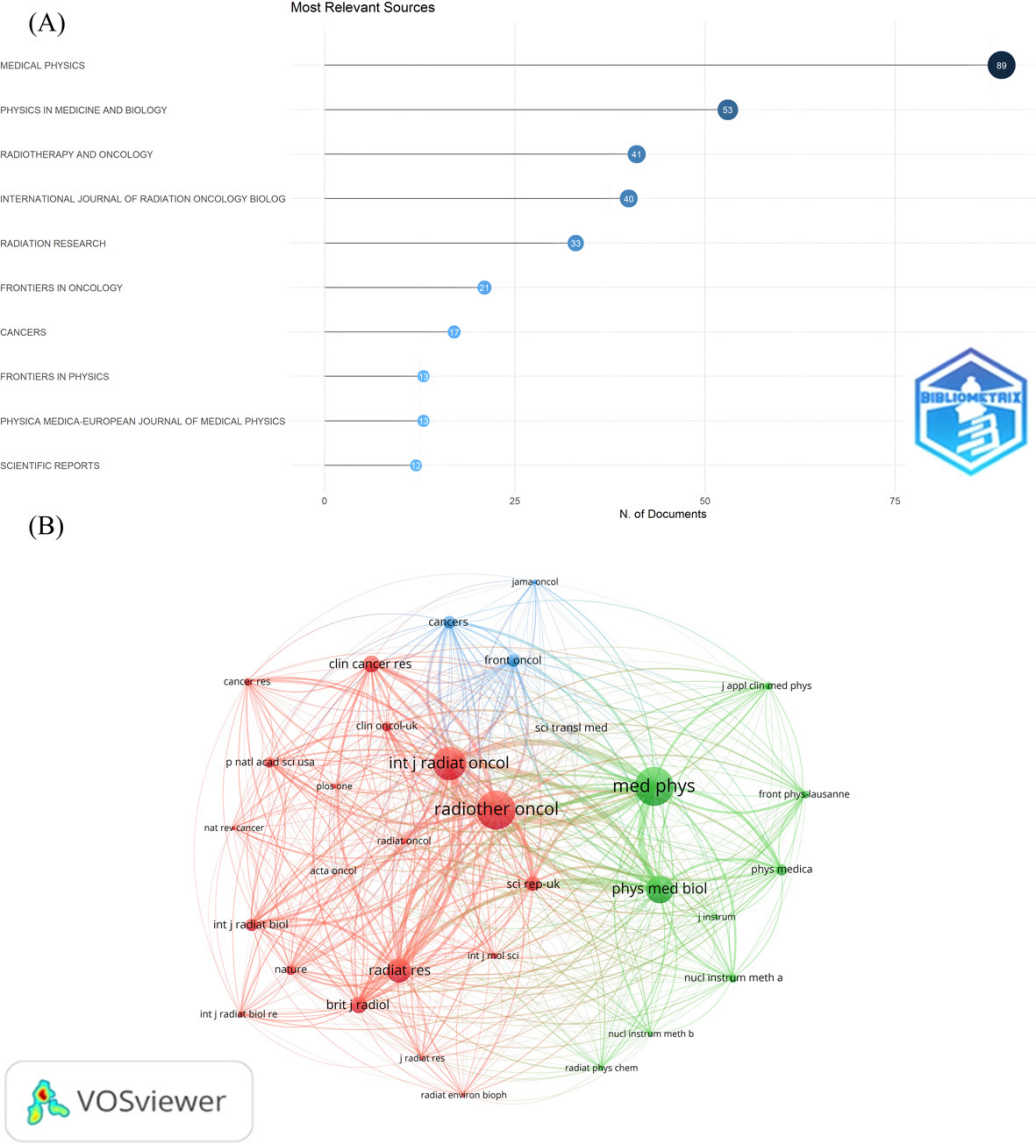
Visual analysis of journal publications. **(A)** The most relevant journals. The Y-axis labels the name of the journal and the X-axis indicates the number of publications in the corresponding journal. **(B)** Journal co-citation relationship mapping. Different colors represent different clusters. Each node represents a journal, and the larger the node, the more co-cited the journal is. The line between the nodes indicates the co-citation relationship between journals, and the thicker the line, the closer the co-citation relationship.

We also conducted a visual analysis of the journals publishing in the FLASH-RT field. The ten journals with the highest number of articles in this area were identified (**Figure 6A**). Medical Physics has the most publications (n=89). Additionally, the top five journals based on citation and co-citation counts are presented in **Table 1**. Radiotherapy and Oncology has the highest number of citations, while Medical Physics leads in co-citations. A co-citation analysis of the journals was also performed (**Figure 6B**). From a total of 2,375 journals analyzed, only those with more than 30 co-citations were included, resulting in 32 journals being classified into three distinct clusters.

**Table 1:** Top five journals for citations and co-citations.

Rank	Top 5 popular journals	Citations (n)	Top 5 cited journals	Co-citations (n)
1	Radiotherapy and Oncology	2945	Medical Physics	2292
2	Medical Physics	1611	Radiotherapy and Oncology	2259
3	International Journal of Radiation Oncology Biology Physics	1535	International Journal of Radiation Oncology Biology Physics	1748
4	Clinical Cancer Research	823	Physics in Medicine and Biology	1310
5	Radiation Research	750	Radiation Research	1056

### 3.5 Citation and co-citation analysis of publications

**Figure 7:**
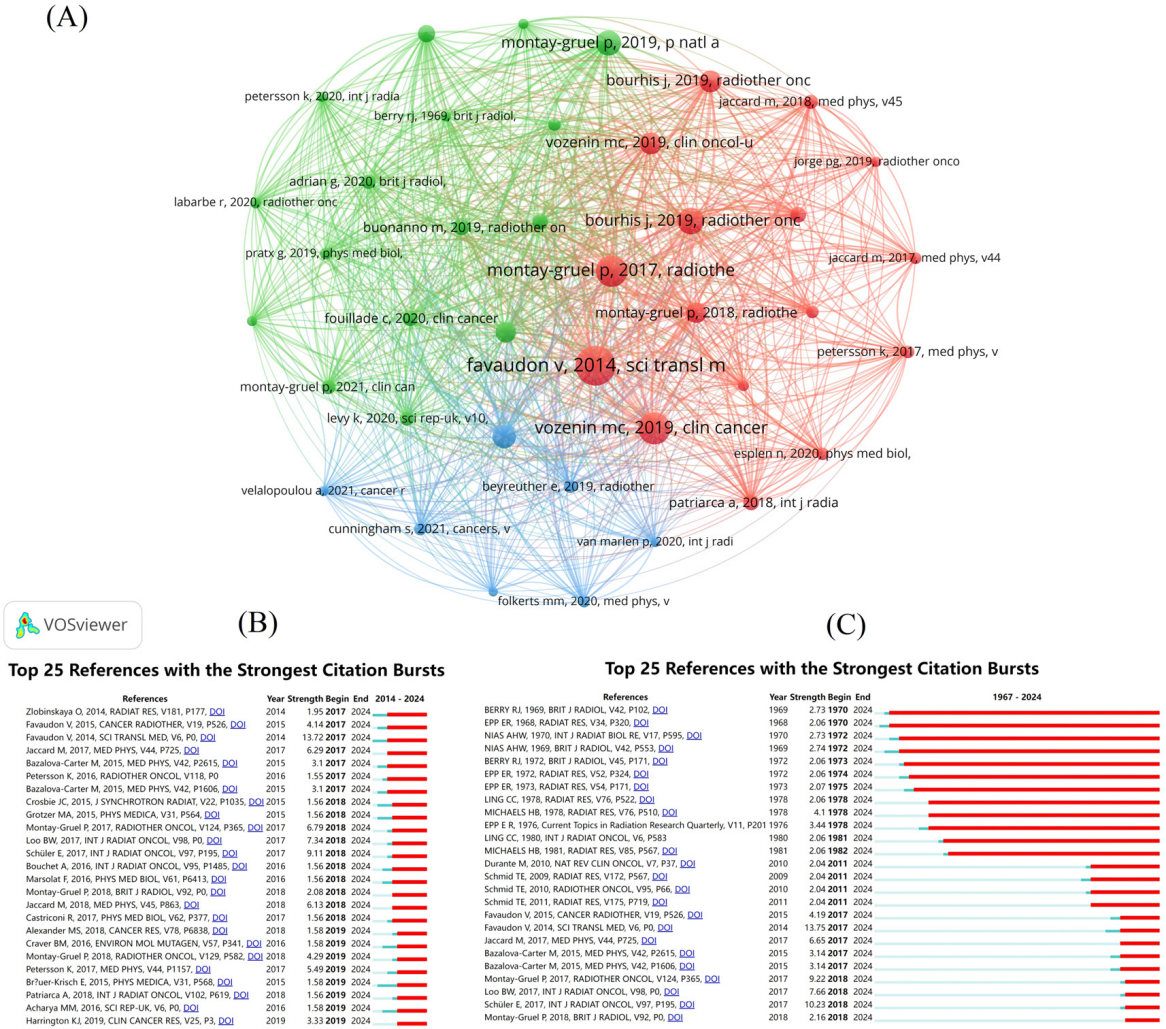
Visual analysis of publications. **(A)** Literature co-citation analysis mapping. Different colors represent different clusters. A node represents a piece of literature, and the larger the node is, the more co-citations the literature has. The line between the nodes represents the co-citation relationship between the documents, and the thicker the line, the stronger the co-citation relationship between the documents. **(B)** Citation explosion graphs for literature from 1967-2024. **(C)** Citation explosion graphs for literature from 2014-2024.

Among the cited literature, we identified the top 20 most frequently referenced documents (**Table 2**). These documents were ranked in descending order based on citation count, and their average citations per year were also calculated. A visual analysis of the co-citation relationships among these documents was conducted (**Figure 7A**). A total of 8,312 documents exhibited co-citation relationships, with 39 documents receiving more than 60 co-citations. These 39 documents were analyzed and categorized into three broad clusters. Additionally, a citation burst analysis was performed, highlighting the 25 documents with the highest citation burst intensity (**Figure 7B**). One prominent document published in 2014, titled “Ultrahigh Dose-Rate FLASH Irradiation Increases the Differential Response Between Normal and Tumor Tissue in Mice”, stands out with the highest number of citations and co-citations. Following this, we conducted another citation burst analysis, focusing on the publication date of this key document (**Figure 7C**). This series of analyses clearly identifies the literature that has significantly influenced the FLASH-RT field, providing guidance for future research. It also helps us determine which documents are frequently used together, serving as a theoretical foundation for subsequent studies.

**Table 2:** The 20 documents with the highest number of citations.

Paper	DOI	Total Citations	Normalized TC
FAVAUDON V, 2014, SCI TRANSL MED (10)	10.1126/scitranslmed.3008973	803	1.82
VOZENIN MC, 2019, CLIN CANCER RES (25)	10.1158/1078-0432.CCR-17-3375	462	2.59
MONTAY-GRUEL P, 2017, RADIOTHER ONCOL (13)	10.1016/j.radonc.2017.05.003	423	1.42
BOURHIS J, 2019, RADIOTHER ONCOL-a (26)	10.1016/j.radonc.2019.06.019	400	2.24
MONTAY-GRUEL P, 2019, PROC NATL ACAD SCI U S A (27)	10.1073/pnas.1901777116	326	1.83
VOZENIN MC, 2019, CLIN ONCOL (28)	10.1016/j.clon.2019.04.001	316	1.77
WILSON JD, 2020, FRONT ONCOL (29)	10.3389/fonc.2019.01563	305	4.33
BOURHIS J, 2019, RADIOTHER ONCOL (9)	10.1016/j.radonc.2019.04.008	300	1.68
DIFFENDERFER ES, 2020, INT J RADIAT ONCOL BIOL PHYS (30)	10.1016/j.ijrobp.2019.10.049	283	4.01
MONTAY-GRUEL P, 2018, RADIOTHER ONCOL (12)	10.1016/j.radonc.2018.08.016	245	1.67
SPITZ DR, 2019, RADIOTHER ONCOL (31)	10.1016/j.radonc.2019.03.028	182	1.02
PATRIARCA A, 2018, INT J RADIAT ONCOL BIOL PHYS (32)	10.1016/j.ijrobp.2018.06.403	180	1.23
BUONANNO M, 2019, RADIOTHER ONCOL (33)	10.1016/j.radonc.2019.02.009	177	0.99
SCHULER E, 2017, INT J RADIAT ONCOL BIOL PHYS (34)	10.1016/j.ijrobp.2016.09.018	171	0.58
SIMMONS DA, 2019, RADIOTHER ONCOL (35)	10.1016/j.radonc.2019.06.006	163	0.91
FOUILLADE C, 2020, CLIN CANCER RES (36)	10.1158/1078-0432.CCR-19-1440	161	2.28
DURANTE M, 2018, BR J RADIOL (37)	10.1259/bjr.20170628	161	1.10
ESPLEN N, 2020, PHYS MED BIOL (38)	10.1088/1361-6560/abaa28	159	2.26
MONTAY-GRUEL P, 2021, CLIN CANCER RES (11)	10.1158/1078-0432.CCR-20-0894	155	4.64
ADRIAN G, 2020, BR J RADIOL (39)	10.1259/bjr.20190702	144	2.04

### 3.6 Visualization and analysis of keywords

**Figure 8:**
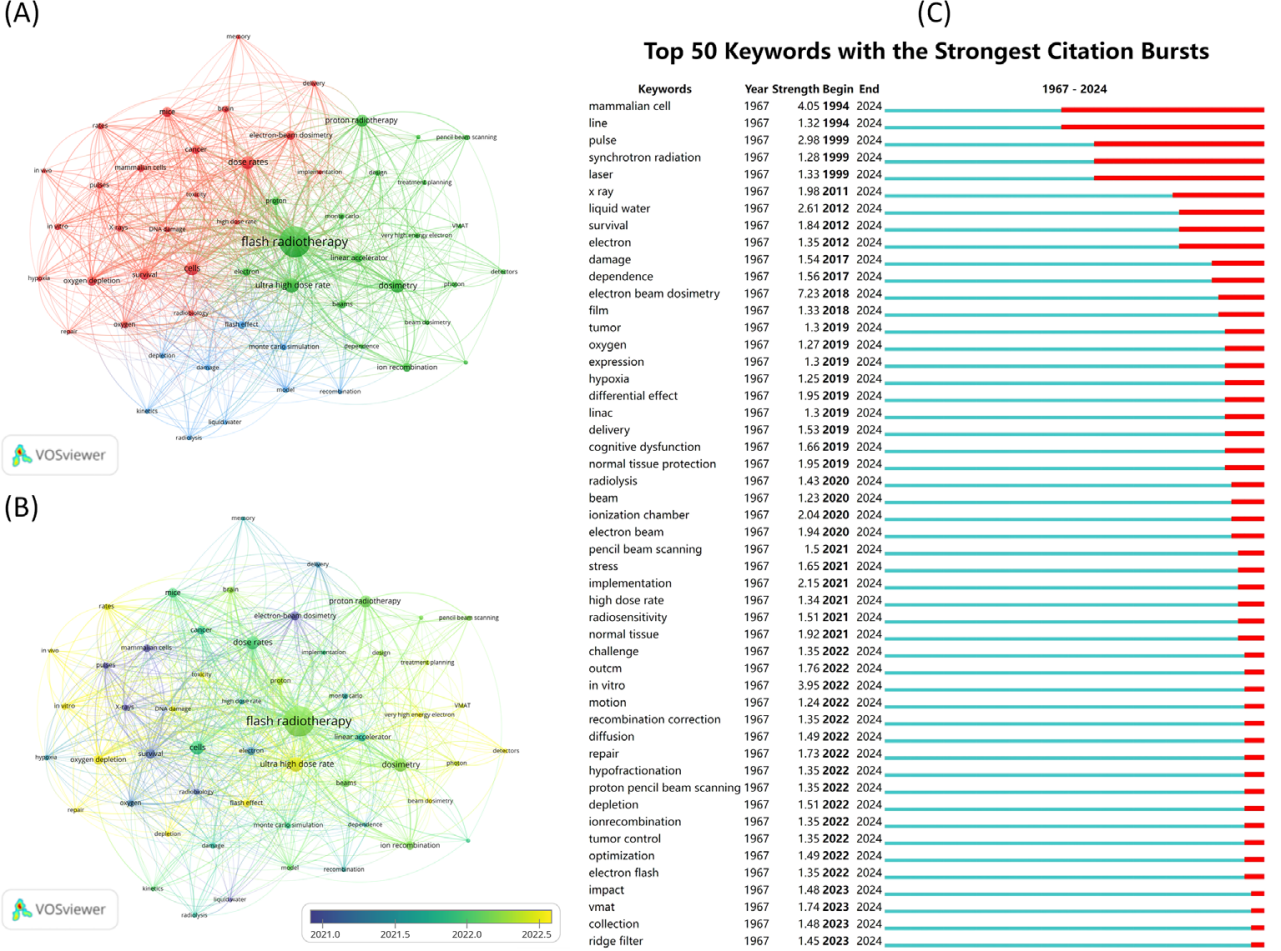
Visual analysis of keywords. **(A)** Keyword co-occurrence analysis. Different colors represent different clusters. Each node represents a keyword, and the larger the node, the higher the frequency of this keyword. The line between the nodes indicates a co-occurrence relationship between the keywords, and the thicker the line, the more frequent the co-occurrence of the two. **(B)** Keyword average year graph. Shows the average year in which keywords appear. **(C)** Keyword eruption chart.

We conducted a visual analysis of the keywords in the included articles. First, we generated a co-occurrence map of the keywords, along with a keyword average age map (**Figures 8A, B**). This analysis focused on 54 keywords, each appearing at least eight times. The keywords were grouped into three clusters to highlight their relationships. The five keywords with the highest co-occurrence counts are “flash radiotherapy” (n=379), “ultra-high dose rate” (n=86), “dosimetry” (n=77), “cells” (n=74), and “dose rates” (n=72). The average age of the keywords reveals shifts in focus over time, with “survival” being replaced in recent years by terms like “flash effect” and “ultra-high dose rate”. Additionally, a burst analysis of keywords was performed, identifying the 50 keywords with the highest burst intensity (**Figure 8C**). The keyword “mammalian cell” exhibited the highest burst intensity.

## 4 Discussion and critical review

### 4.1 In-depth analyses of the results sections

In the previous section, we observed a growing body of research on FLASH-RT by scholars in recent years. Among the 461 publications included in our analysis, the overwhelming majority were original articles (n=417). A substantial proportion of these publications appeared in high-impact journals within the fields of radiation oncology and medical physics. Unlike previous reviews that provided in-depth summaries of specific FLASH-RT topics, this review uses bibliometrics to offer a comprehensive overview of the entire field. Existing bibliometric analyses in radiation therapy have involved FLASH-RT. Song, G et al. highlighted proton FLASH as an upcoming research hotspot, which aligns with our findings (18). Our study further enriches this discourse by providing a comprehensive examination of FLASH-RT’s development and status. We employed bibliometric analysis to assess the full spectrum of research methods used in these publications, yielding noteworthy results. These results can largely be attributed to three key factors: the mechanisms underlying the FLASH effect remain unclear and are the focus of extensive research; the devices and equipment utilized for FLASH-RT are not widely accessible and have been rapidly evolving in recent years and the clinical transition of FLASH-RT presents a challenge that awaits resolution (40–42).

Since 2017, FLASH-RT publications have experienced exponential growth, likely attributable to several key factors. The FLASH concept was first proposed in 2014 (10). Its impact was immediate, yet related research efforts took time to develop. The middle three years were occupied with experiment design, equipment preparation, and the writing and approval of papers. The 2017 publications that observed the FLASH effect marked a turning point (13). The successful replication of the FLASH effect and its scientific potential are the primary reasons for the subsequent surge in publications.

While 40 countries contributed to this field, the predominant countries accounted for the vast majority of publications. The United States leads in FLASH-RT publications due to government funding, exemption policies, equipment for generating UHDR beams, and the FLASH Alliance. The National Institutes of Health, the largest global public funder of biomedical research, supports many scientific projects. In June 2022, the U.S. Food and Drug Administration approved the Investigational Device Exemption for the FAST-02 human clinical trial, advancing FLASH-RT research. The U.S. also has equipment infrastructure as the largest proton therapy market with the most operating proton centers. American firms, such as Varian, offer crucial technological support. Varian’s ProBeam proton therapy system was designed with the capability to deliver UHDR proton beams incorporated from the outset. Varian formed the FlashForward consortium in 2018, uniting thirteen institutions on UHDR proton therapy for cancer. Similar alliances exist in Europe, like the FLASHKNiFE alliance announced by PMB, bringing together top European institutions. These alliances promote international cooperation. Concurrently, we posit that inter-institutional co-authorship is somewhat shaped by inter-country cooperation. We observe that in the institutional co-authorship graph, institutions within the same cluster originate from different countries, further illustrating the concept of national co-authorship. For instance, the institutions in the red cluster represent seven different countries: Switzerland, the United Kingdom, the United States, Sweden, Italy, Denmark, and Germany.

The co-authorship clusters are also characterized by a tendency for different clusters to focus on specific types of FLASH; the more similar the types, the closer the clusters are to each other, resulting in a higher degree of integration. Vozenin, Marie-Catherine’s cluster concentrates on the biological effects of FLASH. Zhang, Rongxiao’s cluster is centered around the mechanisms underlying FLASH effect. The cluster related to Zhang, Rongxiao emphasizes the mechanisms of the FLASH effect. The co-citation relationships among the authors highlight the overlap of their research interests and their influence within the FLASH-RT field. An author’s co-citation count is influenced by the number of high-impact articles and the total number of publications in their related field, and there exists a positive correlation between the two. We analyzed the publication patterns of the three most influential authors in FLASH-RT research. They entered the field early and gained prominence through high-impact reviews, experiments observing the FLASH effect, or new mechanisms that caused widespread repercussions.

Several factors contribute to the findings of the co-citation analysis of the journals. The journals in the red cluster feature articles on FLASH research with a broader focus, exemplified by Radiotherapy and Oncology (n=2259) and International Journal of Radiation Oncology Biology Physics (n=1748). Conversely, the journals in the green cluster primarily focus on research devices and techniques related to FLASH. Medical Physics (n=2292) and Physics in Medicine and Biology (n=1310) are among the core journals, contributing significantly to the volume of articles with high co-citation counts. In contrast, the blue section is dedicated to examining the feasibility of FLASH applications for treating specific clinical diseases, though it currently holds less influence in the FLASH-RT field.

The five keywords with the most co-occurrences center around the radiotherapy domain, suggesting that the FLASH technique, characterized by UHDR, is broadly acknowledged within this field. The remaining keywords, concerning both biological mechanisms and devices, emphasize the implementation of FLASH-RT in clinical practice. The term “mammalian cell” exhibits the highest explosive frequency, attributed to continuous preclinical and clinical trials conducted from the 1970s to the present day.

### 4.2 Biological mechanisms of FLASH-RT

So far, the biological mechanism of FLASH-RT is not clear, but some popular viewpoints have emerged. In the previous keyword clustering analysis, we can see “hypoxia” (n=12), “depletion” (n=12), “oxygen” (n=22), “kinetics” (n=11) and “oxygen depletion” (n=41). This is closely related to the oxygen depletion hypothesis of FLASH-RT. The frequent occurrence of related keywords depends on the controversy and discussion caused by this hypothesis.

As early as 1959, studies revealed that increasing the radiation dose rate could diminish the radiation susceptibility of Salmonella and enhance its survival (43). Subsequent experiments on mammalian cells have yielded similar results, with MILL et al. demonstrating that mammalian cells can repair sub-lethal DNA damage following irradiation at UHDR (44). Early researchers postulated that the decreased radiosensitivity induced by high dose rates was associated with a low oxygen environment (45).

The oxygen depletion hypothesis was the first proposed mechanism for the FLASH effect (28). It initially gained traction due to two factors. First, *in vitro* cell experiments from the last century directly implicated oxygen in radiation responses. Second, it aligns with traditional radiobiological views that hypoxic cells or tissues are more radiation-resistant (39, 46). While this hypothesis initially explained the FLASH effect, numerous studies have since shown it cannot fully account for the phenomenon. Hu et al.’s computer model demonstrated that the hypoxic environment from UHDR irradiation of normal tissues does not reach radiation-resistance levels (47). Moreover, under the same dose conditions, oxygen consumption by tissues from UHDR irradiation is even less than from conventional radiotherapy (48, 49). Research on reactive oxygen species, free radical recombination, and tissue antioxidant systems also challenges the oxygen depletion hypothesis (50, 51). The interaction mechanisms between particle orbits proposed by researchers further support these alternative theories (52, 53). Collectively, these findings suggest that oxygen depletion is just one factor contributing to the FLASH effect.

The immune hypothesis presents another widely accepted perspective on the biological mechanism of FLASH-RT. Although its number of relevant studies is far less than that of the oxygen depletion hypothesis, some results have been achieved. This hypothesis posits that UHDR elicit a distinct immune response within the immune system, subsequently triggering the FLASH effect. In 2020, Jin et al. were pioneers in proposing the explicit hypothesis that UHDR significantly diminishes the cytotoxic effects on circulating immune cells (54). The significance of the inflammatory response in the FLASH effect has been acknowledged in relatively early research (55). Additionally, there have been advancements in studies that specifically target immune cells within the circulatory system. The researchers employed two distinct modeling approaches to examine the effects of continuous partial body irradiation and pulsed irradiation on the levels of surviving blood lymphocytes. They concluded that human blood lymphocyte populations recovered more rapidly from continuous partial irradiation at UHDR compared to conventional dose rates (56, 57). Research on the immunological hypothesis of FLASH-RT is limited, with few comprehensive reviews such as high-quality meta-analyses or biosignal analyses. The number of studies on immunological mechanisms is small, and several critical issues need addressing. Previous research has not clearly identified the specific immune response mechanisms activated by UHDR irradiation. Given that only a small fraction of immune cells are in the circulating blood pool, the mechanism by which this triggers the FLASH effect remains unclear. The FLASH effect induced by UHDR irradiation should be clearly linked to irradiation parameters and the quantity of irradiated immune cells. Resolving these issues will deepen the impact of the immunological hypothesis.

Beyond the two primary hypotheses, there are additional perspectives. For instance, one question is whether the tumor microenvironment influences the FLASH effect (58, 59). Collectively, the exploration of biological mechanisms associated with FLASH is thorough and profound. Although new questions continually emerge from the latest research findings, this is an essential process for the future advancement of FLASH-RT.

### 4.3 Device basis for the realization of UHDR

In order to attain UHDR for application in tumor therapy, appropriate device support is crucial. Devices can be categorized into four types according to the radiation type: electrons, protons, X-rays, and heavy ions. Combined with the results of the bibliometric analysis, the current status of the development of FLASH-RT for the four ray types is different.

#### 4.3.1 Current status of development of electronic FLASH-RT

The keywords “electron” (n=26), “electron-beam dosimetry” (n=37) and “very high energy electron” (n=12) are related to electron FLASH-RT. Evidently, Electronic FLASH-RT has evolved more rapidly and there are more studies on it.

The availability of UHDR the electron-beam (e-beam) surpasses that of the other three radiation types, making their transition from preclinical experiments to clinical applications a logical priority. UHDR electron beams can be generated using specialized miniaturized accelerators or modified medical linear electron accelerators (60, 61). In early 2014, the Oriatron eRT6 linear accelerator, developed by PMB-Alcen, was installed at the University Hospital of Lausanne. This accelerator fulfills the demand for UHDR and high precision radiotherapy equipment for FLASH-RT and represents the first model specifically designed for e-beam FLASH-RT (62). Following this, SIT Sordina developed the ElectronFLASH accelerator, specifically dedicated to research in electron FLASH-RT (63). Modifications and upgrades to existing clinical accelerators can also effectively yield the UHDR e-beam, as illustrated by Garty et al.’s adaptation of a decommissioned Varian Clinac to deliver such beams (64). Successful modifications were also made to two clinical accelerators produced by Medtec (Elekta Precise, Elekta AB) (65). This year, Sloop et al. proposed a design approach that would allow Clinacs to switch between conventional and UHDR modes (66). The enhancement of the classical e-beam accelerator Mobetron has also shown progress in the delivery of the UHDR e-beam (67).

Unfortunately, the penetration depth of the e-beam is quite limited, which may constrain their future applications. Intraoperative radiotherapy represents a promising development for e-beam FLASH-RT, allowing for e-beam therapy in suitable cases of breast, rectal, and pancreatic cancers (68). Very high energy electron (VHEE) beams show potential for treating deep tumors, but they encounter significant challenges (69). As of now, there are no VHEE beams devices available for clinical application, and related studies primarily utilize Monte Carlo techniques for beam modeling (70).

Accurate dose measurement is crucial for the advancement of radiotherapy. The frequency with which the term “detectors” (n=10) appears reflects the progress of FLASH-RT in this area. Initially, Gafchromic EBT-XD films and Advanced Markus parallel plate ionization chambers were employed for the dosimetry of electron beam FLASH (71, 72). Following this, researchers have developed several new devices for e-beam FLASH dosimetry, particularly including: e-beam current transformers, silicon carbide detectors, and diamond detectors (73–75). In developing new equipment, existing dose and detection tools are sometimes employed to assess the accuracy of the new devices in dose monitoring. Gafchromic EBT-XD film and flashDiamond detectors are utilized as comparison standards in the development of new devices (76, 77).

#### 4.3.2 Current status of proton FLASH-RT

Proton radiotherapy is an advanced technique characterized by its unique “Bragg peak” dose deposition, which maximally delivers doses to tumor tissue while minimizing exposure to normal tissues (78). Due to the unique physical properties of proton beams, it is anticipated that they will be capable of treating deep tumors in the future, thereby bridging the gap left by electronic FLASH-RT. In clinical applications of proton radiotherapy, achieving complete tumor coverage requires the diffusion of the original Bragg peak to create an Spread-out Bragg Peak. “Proton” (n=27), “proton radiotherapy” (n=57) and “pencil beam scanning” (n=13), the frequency of these keywords related to proton FLASH-RT reflects its rapid development and the large number of related studies.

In 2020, numerous studies on proton FLASH-RT were conducted. Diffenderfer et al. developed a novel radiotherapy device that employs double-scattered protons to deliver FLASH proton beams guided by computed tomography, and conducted dosimetric verification (30). Darafsheh, A et al. laid the groundwork for preclinical studies by modifying the clinical Mevion HYPERSCAN(R) synchrotron to deliver UHDR proton beams (79). Pencil beam scanning (PBS) is a high-precision, low-side-effect, personalized proton therapy technology with extensive applications and significant therapeutic potential, capable of generating qualified UHDR proton beams (80, 81). Folkerts et al. devised a method for calculating proton field dose rate distributions for PBS, further advancing the research and potential applications of PBS FLASH-RT. Following their research, there has been a continuous stream of enhancements to the original technique along with the creation of new methods (82). In 2021, Nesteruk et al. adapted a clinical PBS to allow for the delivery of proton beams with dose rates ranging from 1 to 9000 Gy/s (83). The synchrotron, essential for the realization of proton FLASH-RT, has also undergone improvements (84, 85). In 2022, Zhang et al. designed and optimized a ridge filter, which is one of the most promising methods for achieving proton FLASH-RT. The ridge filter is an innovative beam modulation device capable of rapidly modulating the proton beam at a single energy, producing a dose distribution closely resembling that of intensity-modulated proton therapy. This rapid beam modulation capability enables proton FLASH-RT to be administered in a very short time, thereby satisfying the timing requirements for FLASH-RT (86, 87). In 2023, Ding et al. introduced a method to attain UHDR proton beams: spot-scanning proton arc therapy combined with FLASH. They provided the first voxel-based treatment that achieves UHDR while maintaining high dose consistency in proton beam therapy. This technique may also streamline the clinical process by removing the necessity for standardized ridge filters (88).

Devices for dose verification and monitoring in proton FLASH-RT are rapidly advancing as well. Faraday cups, capable of dosimetry and characterization of beam properties, provide real-time monitoring and feedback and are unaffected by dose rate; thus, they are anticipated to be used as dose verification devices for proton FLASH-RT (89, 90). High-resolution 2D transmission ionization chambers have been validated for monitoring dosimetric parameters related to the proton penumbral beam under FLASH conditions (8, 91). Besides the two types of dose monitoring equipment noted above, scintillator detectors, films (EBT3, EBT-XD and OC-1), parallel plate ionization chambers, and amorphous silicon detectors have also shown distinct advantages and potential (92–95).

#### 4.3.3 Current status of development of X-ray FLASH-RT

X-rays are currently the most commonly used type of radiation in radiotherapy. Furthermore, X-rays have been shown to induce the FLASH effect (12). The keywords “X-rays” (n=19) and “Volumetric-Modulated Arc Therapy (n=8)” are related to photon radiotherapy. It can be seen that the number of studies related to X-ray FLASH-RT is low compared to electrons and protons.

Several platforms offer UHDR from high-energy X-rays. However, most are still in the research phase or lack relevant biological evidence (96, 97). A well-established platform for X-ray FLASH research, referred to as “partner”, is described below. In 2022, Gao et al. assessed the partner platform developed at the Chengdu Terahertz Free Electron Laser Facility. They achieved promising results, confirming that the partner platform can deliver UHDR X-rays and induce the FLASH effect (98). The platform was subsequently re-evaluated by Yiwei Yang et al., yielding consistent results. Following dose verification using devices such as the EBT3 radiochromic film and a fast current transformer, the experimental platform demonstrated the ability to deliver high-energy X-rays at dose rates exceeding 1000 Gy/s.

The primary types of relevant dose detection equipment include silicon-based sensors, scintillator detectors, color-changing films, and thermoluminescent dosimeters (99–101). Effective thermal management of the X-ray tube is essential because of the high current needed to produce UHDR high-energy X-rays (102). Besides the aforementioned related devices, research on collimators has also been undertaken. The experimentation and optimization of decoupled ring collimators and GRID collimators have yielded potentially effective engineering solutions for X-ray FLASH-RT, while also identifying several parameters that may influence the dose rate (103, 104).

#### 4.3.4 Current status of development of heavy ion FLASH-RT

As a state-of-the-art tumor treatment technology in the 21st century, heavy ion radiotherapy has seen significant development and application globally in recent years. As heavy ion radiotherapy is an emerging technology, the associated equipment base is relatively underdeveloped. But there has been some progress in research related to heavy ion FLASH-RT (105).

In 2023, Yagi et al. proposed that UHDR carbon ion beams could be generated using a medical synchrotron, while also highlighting the potential risk of damage to monitoring devices (106). In 2024, Lang et al. developed a FLASH ionization chamber designed for dosimetry of carbon ion FLASH-RT under low-pressure conditions, using a Faraday cup to confirm its dose-rate dependence (107). A specialized dose detector for UHDR carbon ion beams was also created, capable of measuring dose, dose rate, and dose profile. This development offers insights for the future advancement of related devices: employing large plane-parallel ionization chambers with small electrode spacing may enable more accurate monitoring of the dose in heavy-ion FLASH (108).

While FLASH-RT equipment is rapidly evolving, its development and clinical application face technical barriers. Triggering the FLASH effect requires numerous specific conditions, many of which differ from conventional radiotherapy parameters. A key requirement is an UHDR (40Gy/s), far exceeding existing clinical radiotherapy equipment capabilities. This poses a severe challenge to current accelerator technology. Achieving such a high dose rate demands extremely high beam intensity and stability from accelerators, as well as precise radiation energy delivery to target tissue in an extremely short time. Operating under these high beam conditions exceeds conventional accelerator capabilities, and also imposes higher demands on accelerator scattering performance (109). Additionally, FLASH-RT has stringent requirements for the fine temporal structure of beam dose delivery. Parameters such as pulse dose rate, pulse duration, pulse interval time, number of pulses, total irradiation time, and average dose rate can all potentially affect the FLASH effect (110). These parameters also challenge dose monitoring devices. Traditional dosimetry equipment cannot be directly applied to FLASH-RT. For example, UHDR beams may cause detector saturation and ion recombination, compromising measurement accuracy (111). Furthermore, detectors must be able to resolve the beam’s time structure. The ability to characterize in real-time the temporal and spatial structure of the transmission beam, and measure physical parameters under UHDR beam conditions, represents new requirements that FLASH-RT imposes on dose detection equipment (112, 113).

### 4.4 Evaluation of relevant preclinical and clinical trials

The advancement of FLASH-RT is fundamentally rooted in extensive preclinical and clinical experiments. In the previous keyword analysis, “cells” (n=74), “mice” (n=41), “mammalian cells” (n=24), “brain” (n=21), “*in vitro*” (n=19), “*in vivo*” (n=9), “memory” (n=9) and other keywords reflect to some extent the clinical transformation of FLASH.

Preclinical experiments in FLASH-RT can be generally categorized into two main types: cellular experiments and live animal experiments. To our knowledge, the cells utilized in preclinical experiments include dried Artemia eggs, tumor cells, normal human lung fibroblasts, zebrafish embryos, clonally derived CHO-KI cells, and cryptic nematode embryos (33, 114–117). Cellular experiments are typically conducted under *in vitro* conditions and are often used to investigate some basic metrics. For instance, researchers often examine whether there are differences in survival rates after irradiation with conventional radiotherapy compared to FLASH-RT, and whether any FLASH effect is observed. Numerous animal types are also suitable for preclinical experiments. In 2019, Vozenin et al. conducted UHDR irradiation on minipigs and cats, yielding evidence of the benefits of FLASH-RT and strongly advocating for further evaluation of FLASH-RI in human patients (25). Subsequently, minipigs and cats have been studied as larger vertebrate models to validate the protective effects of FLASH (118). In 2021, Konradsson et al. conducted a study on treating superficial malignancies using the FLASH e-beam in canine patients, assessing the feasibility and safety of the approach (119). Following this, Gjaldbaek et al. also investigated the efficacy of FLASH-RT on canine tumors. There is no doubt that among all animal models, the mouse is the most commonly used and effective model. Researchers have noted protective effects on brain tissue, lungs, skin, heart, esophagus, and intestines in mice subjected to UHDR (120–124). Similar design thinking and depth of exploration are evident in mouse experiments. For instance, in 2020, Allen et al. utilized a mouse model to elucidate the protective effects of FLASH-RT on brain tissue. They propose that FLASH-RT may safeguard the vascular system in mice without causing damage to the blood-brain barrier (125). In 2021, Montay-Gruel et al. employed dose-fractionated therapy to simultaneously validate the neuroprotective effects of FLASH-RT in mice and optimize tumor therapy (11). Mouse experiments likewise reveal potential future clinical applications. Radiotherapy is a critical treatment for glioblastoma, and in 2022, Liljedahl et al. conducted experiments on mice, concluding that FLASH was equally effective in fully immunocompetent animals with glioblastoma, a finding that could be advantageous for glioblastoma treatment (126).

Existing FLASH-RT clinical studies have yielded relatively positive results. The treatment of the first patient receiving FLASH-RT was reported in 2019. Bourhis et al. administered electron beam FLASH-RT at the University Hospital of Lausanne for a 75-year-old patient with cutaneous lymphoma. The results were encouraging: the patient exhibited a sustained anti-tumor response and experienced fewer radiation-related side effects than initially anticipated (26). This human trial confirmed the feasibility and safety of FLASH-RT for clinical applications. It was followed by two clinical trials involving proton FLASH-RT. In 2023, Mascia et al. conducted a non-randomized trial of palliative FLASH-RT for limb bone metastases in participants treated at the Cincinnati Children’s/University of California Health Proton Therapy Center. In this non-randomized trial, metrics concerning clinical workflow, treatment efficacy, and safety data demonstrated that UHDR proton FLASH-RT is clinically feasible (127). In 2024, Daugherty et al. evaluated treatment toxicity and pain relief in study participants with painful sternal metastases who were treated with FLASH-RT, along with assessing workflow metrics in a clinical setting (128). Undoubtedly, the clinical research on FLASH-RT is confronted with challenges. The scarcity of clinical studies and slow progress can be attributed to several factors. The biological mechanisms underlying FLASH-RT remain unclear, and the lack of specialized equipment further hampers clinical investigation. Selecting appropriate patients based on tumor characteristics such as type, location, and stage is also a formidable task (14). Although preclinical studies have yielded promising results, the substantial anatomical and physiological differences between animal models and human models cast doubt on the direct applicability of these findings to clinical safety and efficacy (129). Moreover, the involvement of high-energy radiation introduces complex ethical and regulatory issues (130). These factors collectively contribute to the certain obstacles faced in advancing FLASH-RT clinical research. In summary, the clinical translation process of FLASH-RT relies on advanced accelerator equipment as well as dose detection devices and treatment planning systems that complement it. At the same time, support from relevant policies also promotes the clinical translation process. Researchers can also consider introducing advanced technologies from traditional radiation therapy (such as image-guided and adaptive radiotherapy) and processes (such as quality assurance) into FLASH-RT. This is beneficial for the development of FLASH-RT.

## 5 Limitation

The data for this study is sourced from the WOSCC database, which does not encompass all the literature in this field. The lack of cross-validation with other datasets introduces risks of both dataset selection bias and overfitting. Therefore, the results may not fully represent the current status of the entire FLASH-RT domain. Related research in this field has advanced rapidly in recent years, and this econometric analysis can only partially reflect the current state of development based on the literature included. Furthermore, as co-citation frequency is time-dependent, high-quality literature published in recent years may exhibit lower co-citation frequencies because of their shorter publication periods, leading to a discrepancy with the actual situation. When employing VOSviewer and CiteSpace for data visualization and analysis, there is no standardized reference for the time division, thresholding, and cropping methods applied to the data, which may introduce bias.

## 6 Conclusion

Our bibliometric analysis of FLASH-RT delves into several key dimensions of the field. We systematically examined publication trends to identify growth patterns and pivotal moments in research output. Our analysis also mapped international collaborations, revealing the global network of institutions and countries contributing to FLASH-RT advancements. We further identified influential journals and authors who have significantly shaped the discourse through high-impact contributions. Additionally, we analyzed keyword evolution to trace shifting research priorities and emerging focal points within the field. Additionally, it examines recent progress in biological mechanisms, equipment, and clinical translation. This analysis aims to offer researchers a comprehensive overview of the field.

## Data Availability

Research data are stored in an institutional repository and will be shared upon request to the corresponding author.
