# Supplementary material for: The evolution of FLASH radiotherapy: a bibliometric analysis
Source: Front Oncol. 2025 May 15;15:1580848. doi: 10.3389/fonc.2025.1580848 (PMC12119588; doi:10.3389/fonc.2025.1580848)
Supplement: Supplementary file 1 [file DataSheet1.docx]

Supplementary Material

# Retrieval strategy

((((TI=(（radiation therapy OR radiotherapy OR radiotherapy treatment OR particle therapy OR proton therapy OR Irradiation OR Proton) AND (FLASH OR UHDR OR Ultra-high Dosage Rate OR Ultra-high dose-rate OR Ultra-High-Dose-Rate OR Ultrahigh Dose-rate OR Ultra-High Dose Rate OR Ultra-High Dose Rates）)) OR TI=(（clinical trials OR clinical translation） AND （flash OR UHDR OR Ultra-high Dosage Rate OR Ultra-high dose-rate OR Ultra-High-Dose-Rate OR Ultrahigh Dose-rate OR Ultra-High Dose Rate OR Ultra-High Dose Rates）)) OR TI=(UHDR OR ultrahigh dose-rate OR Ultra-high Dosage Rate OR “FLASH effect” OR “FLASH-RT” OR “FLASH-IMPT” OR FLASH dose rate OR FLASH dose delivery)) OR TI=(（FLASH machine）AND LINAC)) OR TI=(（FLASH OR UHDR OR Ultra-high Dosage Rate OR Ultra-high dose-rate OR Ultra-High-Dose-Rate OR Ultrahigh Dose-rate OR Ultra-High Dose Rate OR Ultra-High Dose Rates） AND electron AND （radiation therapy OR radiotherapy OR radiotherapy treatment OR particle therapy OR Irradiation OR dose delivery OR LINAC）)
